# Impedance sources (Z sources) with inherent fault protection for resilient and fire-free electricity grids

**DOI:** 10.1038/s41598-024-53452-y

**Published:** 2024-02-06

**Authors:** Fang Zheng Peng

**Affiliations:** Florida State University, Tallahassee, USA

**Keywords:** Energy science and technology, Engineering

## Abstract

Modern societies would not survive without electricity and at the same time electrical faults could cause and have caused many catastrophes—mainly deadly fires—to our societies. There are two types of electricity sources: the voltage source such as generators, charged batteries and capacitors, and the current source such as charged inductors, current-regulated rectifiers, and superconducting magnetic energy storage. An “ideal” voltage source—that is often-sought-or-intentionally engineered—generates a constant voltage irrespective of its load current, and an “ideal” current source injects a constant current irrespective of its load voltage. However, two problems exist: (1) voltage or current sources do not represent many emerging natural/renewable energy sources such as wind turbine generators, photovoltaic cells, and fuel cells, whose output voltage and current are strongly dependent on each other, and (2) a short-circuit fault to an artificially-made and controlled “ideal” voltage source or an open-circuit fault to an “ideal” current source can cause catastrophic failures of the source itself and its surrounding circuits due to large (theoretically infinite) short-circuit current or open-circuit voltage. Here we introduce an impedance source concept to represent, characterize, and model those electricity sources whose output voltage and current are strongly dependent on each other. First, we found that many electric sources with no feedback (or active) control of their output voltage and/or current are a natural impedance source with inherent fault protection at short-circuit or open-circuit faults. Second, any electrical source can be artificially controlled to mimic a natural impedance source. Finally, we show how to apply natural impedance sources and nature-mimicking artificially-controlled sources to the electricity grid—the most complex machine ever made by human beings—to realize electricity grids that are naturally stable, self-protected against electrical faults, and resilient to natural and human-made events.

## Introduction

**Figure 1:**
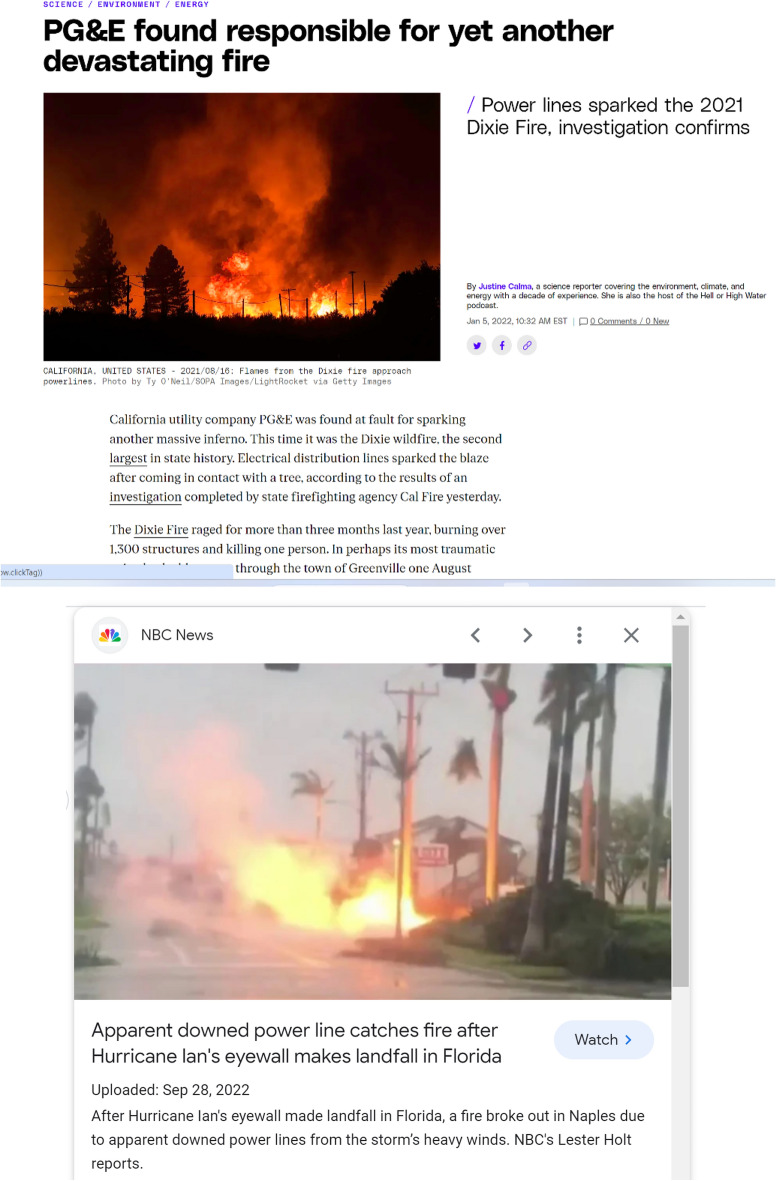
Top: Report photos of the 2021 California Dixie fire sparked by power lines. Bottom: NBC News report shows caught-on-video images of fires ignited by downed power lines after Hurricane Ian’s eyewall made landfall in Florida in September 2022.

The recent (August 2023) Maui wildfires are the deadliest in the U.S. in more than 100 years, according to the National Fire Protection Association and reportedly it was caused by power lines downed by wind storms and their consequential electrical faults and fires just like many other confirmed cases^1–3^. For example, the huge 2021 Dixie Fire in California^4^ was sparked by power lines and the case confirmed after a year-long investigation, as shown in the top of Fig. 1. It is a well-known fact that power lines downed to the ground by wind storms or downed tree branches over power lines can cause fires because these conditions create short-circuit faults with large overcurrent that overheats everything in the path and/or open-circuit faults with huge overvoltage causing electrical arc flashes that reach a temperatures of 35,000°F—hotter than the surface of the sun. Consequentially, they spark fires, as shown in the bottom of Fig. 1 as another example caught on video camera and reported by NBC News.

Short-circuit or open-circuit faults in power grids are inevitable because of natural disasters, equipment failures, and human errors. However, do electrical faults inevitably generate large overcurrent or huge overvoltage? What are the root causes of large short-circuit overcurrent and/or open-circuit overvoltage? Is it possible to re-make/re-engineer our electricity power grids self-protected, fault-current/fault-voltage limited, and therefore resilient to natural and human-made disasters? The purpose of our research carried out and, therefore, of this research article as a whole is to answer these fundamental questions.

In order to answer the above questions, we need to go back to examine today’s power grid—which consists of four basic parts or subsystems: generation, transmission, distribution, and end-use consumption. Starting from the generation or power sources first: we look into how we have been engineering the power sources since the beginning of power grids over a century ago and their resultant and inherent properties.

Traditionally, there are two types of electricity sources engineered: voltage and current sources. Both voltage and current sources are well known in electrical and electronic engineering that includes many academic disciplines for signal, power, and energy circuits and systems^5,6^.

**Figure 2:**
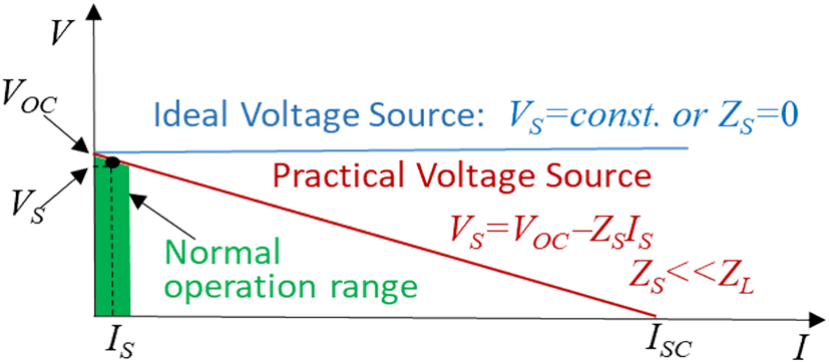
V–I characteristics of a voltage source.

An ideal voltage source as shown in Fig. 2 generates a constant voltage irrespective of current drawn by its load: *V*_*S*_ = *constant*. Therefore, it is clear that an ideal voltage source cannot have a short circuit, which would draw large (theoretically infinite) current and cause damages to the source itself and equipment in its circuit. For a voltage source, no load means an open circuit or zero load current. A more practical voltage source can be regarded as an ideal voltage source with a low series (or internal) resistance for a DC source such as a charged battery, or a low (internal or source) impedance for an AC source such as a generator or grid source. For generality, we use term “source impedance” in this paper because of two considerations: 1) “impedance” has a broader definition than resistance and is valid for both DC (zero frequency) and AC sources, and 2) a “source” can be naturally inherent or artificially made and controlled. Therefore, “source impedance” means an internal (or inherent) impedance in a natural source or by artificially made and controlled.

A typical voltage source’s impedance, *Z*_*S*_ is very low, typically 1/10th to 1/100th as compared to its load impedance, *Z*_*L*_ (that is, *Z*_*S*_* « Z*_*L*_), thus having little voltage drop within the source at any normal operating point (*V*_*S*_, *I*_*S*_) as shown in the green shaded area of Fig. 2. Therefore signal, power, or energy lost or wasted in the source is much less as compared to what is received by the load. In another word, the source voltage, *V*_*S*_ is very close to its open-circuit or no-load voltage, *V*_*OC*_. However, the short-circuit current, *I*_*SC*_ would be far greater (10× to 100× higher) than its normal operating current. The vast white area from the rated current value to short-circuit current, *I*_*SC*_ is not operable and should be avoided because of potential overcurrent damage.

**Figure 3:**
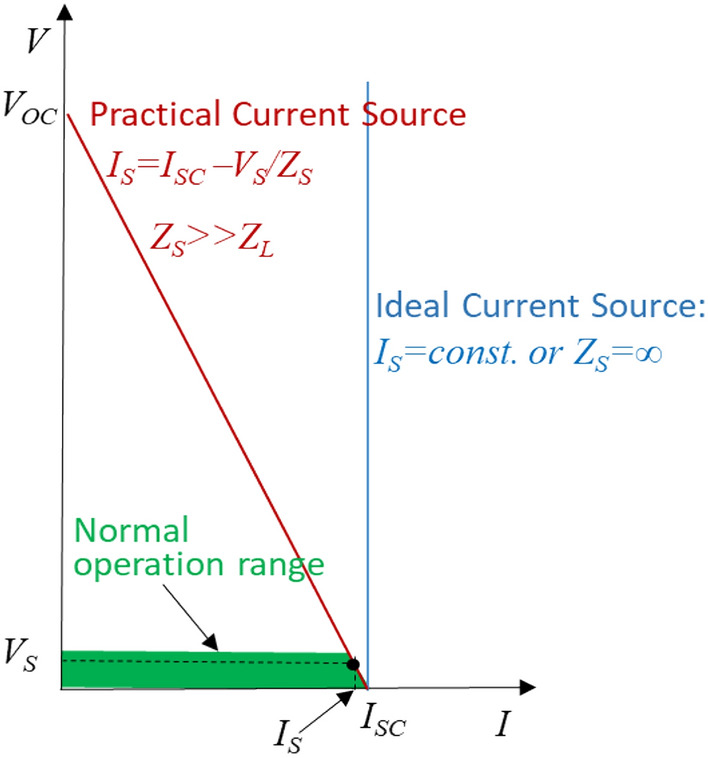
V–I characteristics of a current source.

An ideal current source as shown in Fig. 3 is a source that injects a constant current irrespective of voltage across its load: *I*_*S*_ = *constant*. For a current source, the higher the load impedance, the higher the terminal voltage across the load gets because of the constant current, hence the higher is the power output. Contradictory to a voltage source, a current source’s no-load condition happens at zero resistance/impedance or short circuit. An ideal current source cannot tolerate an open circuit because it would drive the terminal voltage to infinity and destroy the circuit. A practical current source has a relatively high source impedance in parallel with an ideal current source, much higher (typically 10× to 100× higher) than its load impedance, that is, *Z*_*S*_* » Z*_*L*_, thus the current remains practically constant over the operating range irrespective of load impedance. The operable area as shown in green shaded area is very small and limited, similar to the case of a voltage source.

Most practical sources of electricity are voltage sources. Some sources e.g. a charged inductor, the secondary of a current transformer can be regarded as a natural current source. A thyristor-controlled rectifier with a large dc inductor powered from a AC voltage source, a superconducting magnetic energy storage system is a practical current source artificially made.

Traditionally, “ideal” voltage and current sources have been sought or engineered to minimize losses and maximize efficiency. However, it is noticeable that the normal operation range of either voltage or current source is limited to a small area, while the vast V–I characteristic area that should be avoided due to excessive overcurrent or overvoltage—caused by a short-circuit fault to a voltage source or an open-circuit fault to a current source, respectively—presents the root cause to electric fires and equipment damages. In practice, protection devices and circuits must be used to safeguard circuits/systems from overvoltage or overcurrent damages of equipment with limited capabilities/successes due to detection delays, slow response time of circuit breakers, communication latencies, and protection coordination issues. In summary, the vast inoperable and inherent overcurrent/overvoltage region of our engineered power sources and protection delays/issues are the root cause of electric fires and resiliency problems.

It is interesting and surprising to note that there are many sources that exhibit a strong interdependency between their output voltage and current, and have an internal source impedance that is naturally close to their intended load impedance (i.e., *Z*_*S*_ ≈ *Z*_*L*_). Thus, they are far away from “ideal” voltage or current source and cannot be simply regarded as a voltage or current source. This type of sources have been neglected in the mechanical, system control, electrical, and electronic engineering across many different academic disciplines. In addition, many artificial sources could be made, engineered, and/or controlled to have a source impedance ideally equal to its load impedance to achieve different purposes such as maximum signal/power/energy delivery, and reliable/resilient operation.

In this article, we present our results and findings, and methods and approaches in the next sections as follows: First, we present the impedance source (Z source for short) concept and explain how a Z source advantageously makes the entire V–I characteristic region operable, safe, and resilient without external protection circuits. Then we show that most natural and artificial electric and electronic sources with no feedback (or active) control of their output voltage and/or current actually exhibit the nature of a Z source, and have fault protection property without fatal destruction when short-circuited or open-circuited. Further, we show that any electrical signal-, power-, or energy-source can be artificially controlled by today’s enabling technologies—micro-electronics, power (or energy) electronics, and system controls—to behave like a natural Z source. This nature-mimicking method and approach do not compromise efficiency while achieving fault protection and resiliency to extreme conditions like short-circuit or open-circuit caused by natural disasters or human-made events. As our ultimate goals, resilient and fault protected electrical signal, power, and energy sources can be realized. Finally, we hypothesize that the Z source concept and the nature-mimicking approach to the electricity grid—the most complex machine ever engineered and made by human beings—will realize naturally stable and resilient electricity grids by making them resistive or resistance-dominant—because a resistive system is naturally balanced, stable, and resilient.

In summary, the major contributions of our research—and, therefore, of this article—include: (1) identification of the root causes of electrical fires and resiliency problems; (2) presentation and establishment of the impedance source concept and models; (3) methodology/approach to re-making/re-engineering electricity power grids self-protected, fault-current/fault-voltage limited—thus resilient to natural and human-made disasters; and (4) informed perspective on future work for the next century power grids.

## Results and findings

### The Z source concept and characteristics

**Figure 4:**
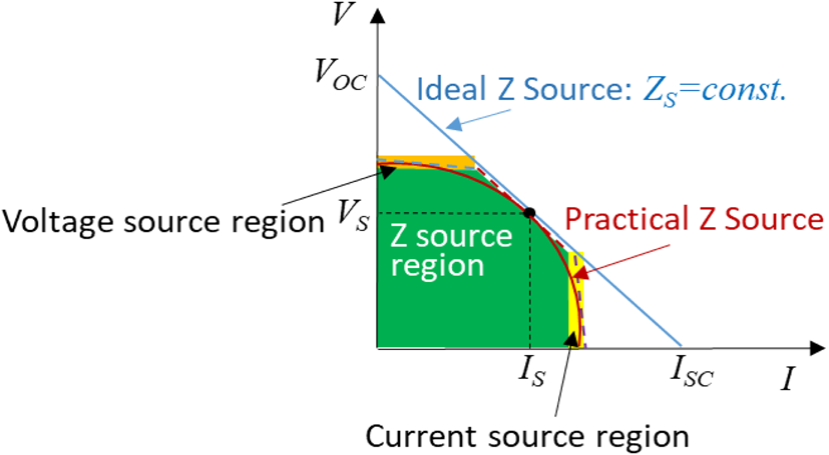
V–I characteristics of an impedance source (Z source).

Figure 4 illustrates the concept and characteristics of a Z source whose output voltage and current are strongly interdependent on each other. An “ideal” Z source exhibits a constant source impedance irrespective of its output voltage and current: *Z*_*S*_ = *constant*. Unlike a voltage or current source, a Z source can be shorted or opened without generating excessive short-circuit current or open-circuit voltage. A practical Z source may behave like the red solid-line curve with further limited open-circuit voltage, *V*_*OC*_ and short-circuit current, *I*_*SC*_, which are even closer to their respective voltage and current ratings. The impedance of a Z source is defined as *Z*_*S*_ = *dV*_*S*_/d(− *I*_*S*_). A linear Z source exhibits a constant impedance equal to *V*_*OC*_*/I*_*SC*_ over the entire operating range, whereas a nonlinear Z source (or a practical Z source as shown in red solid-line curve) a variant impedance dependent on its operating points. Such a practical, nonlinear Z source can be linearized into three pieces as illustrated in the blue, red, and purple dashed lines and their respective regions: voltage source, Z-source, and current source. The Z source region (green area) is the normal operation region, while the voltage-source region (orange area) and current-source region (yellow are) act like a voltage and current limiter.

**Figure 5:**
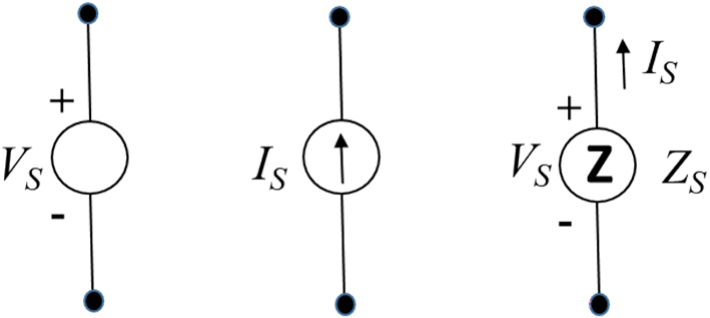
Symbols for an ideal voltage, current, and impedance source.

In summary, a Z source as the third type of electricity sources has the following interesting and distinct features: (1) unlike a voltage source it can be shorted without producing a large short-circuit current, (2) unlike a current source it can be opened without causing a large open-circuit voltage, and (3) its source impedance is comparably close to its load impedance. These three types of electricity sources (their circuit symbols/representations are shown in Fig. 5)—each has its own distinct characteristics and features—together cover the entire spectrum of sources. As presented in the following sections, most renewable energy sources are a natural Z source by themselves or an artificial one by engineering.

### Examples of natural and artificial Z sources

We refer a Z source that has no feedback control of their output voltage and/or output current as a natural (or passive) Z source, and that has feedback control as an artificial (or active) Z source. In this section, we present our findings of some natural and artificial Z sources.

#### Natural (or passive) Z source example I: wind turbine generators

**Figure 6:**
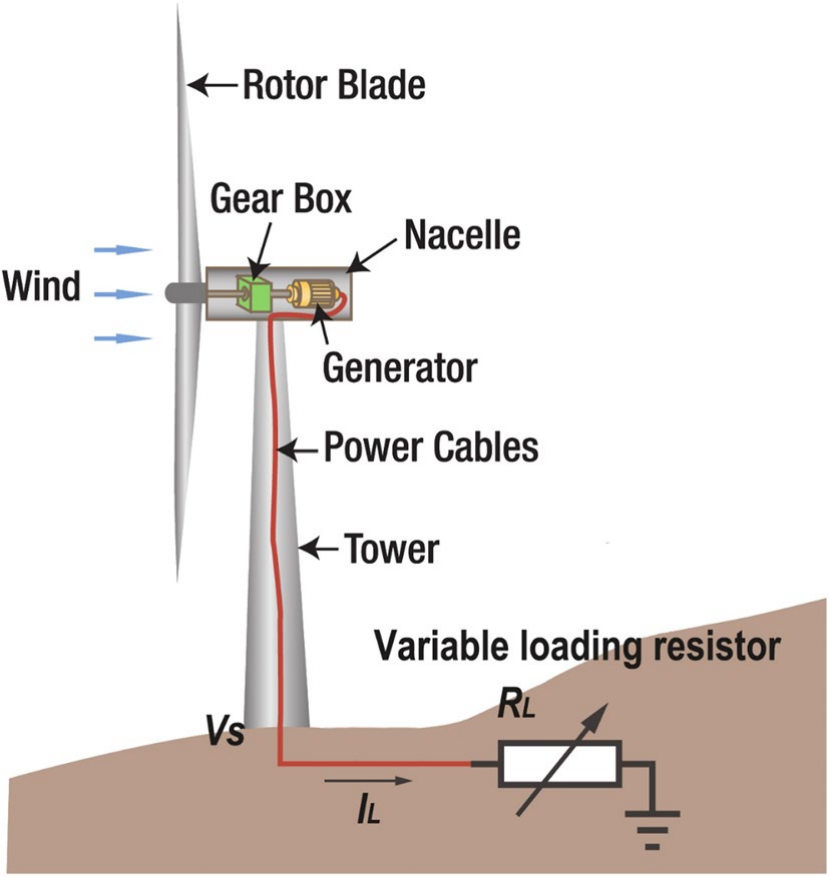
An open-loop wind turbine PM generator feeds a resistive load, *R*_*L*_.

**Figure 7:**
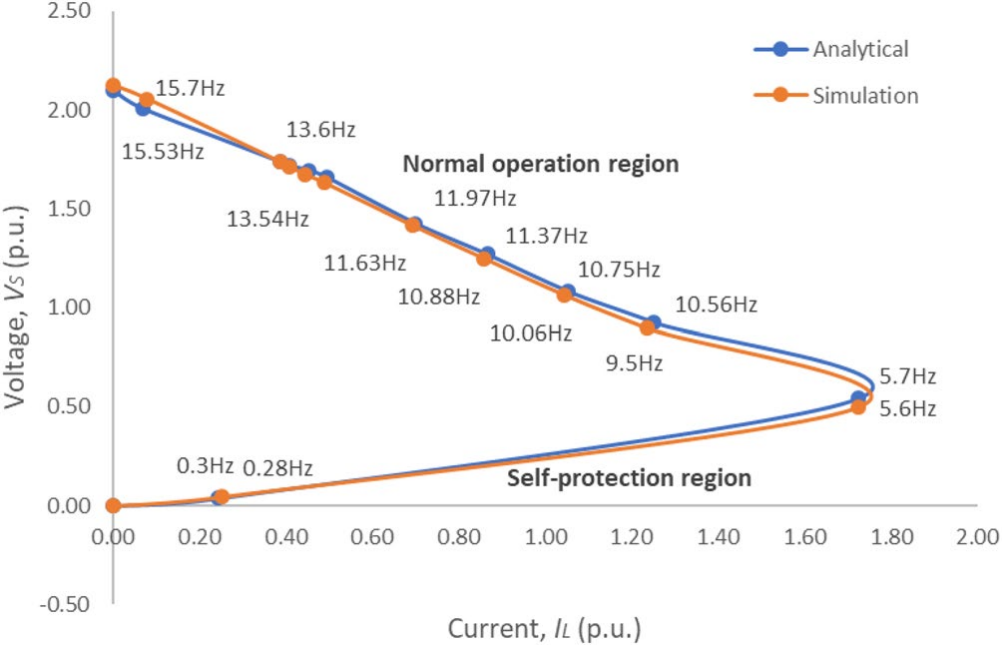
Generator output voltage-current (V–I) characteristic curves.

Consider a wind turbine generator with neither artificial mechanical control (such as pitch angle control) of the turbine nor artificial electrical control of the generator’s output voltage, current, or power, feeding a variable load resistor, *R*_*L*_ as shown in Fig. 6. Figure 7 shows the output voltage versus load current (V–I) characteristics obtained by theoretical analysis and simulation of a 2-MW wind turbine generator, whose parameters are shown in Table 1. The interesting “Big-nose” V–I characteristic curve shows that the output voltage decreases as the load increases (or the load resistance, *R*_*L*_ decreases) from its open circuit (*R*_*L*_ = ∞) voltage linearly down to the “Big-nose” tipping point, behaving just like a Z source. After the “Big-nose” tip point, a further attempt of increasing load (or decreasing *R*_*L*_) actually causes a sharp decrease in the generator output voltage, which naturally reverses the load current trend and reduces load current surprisingly all the way down to zero as the load resistance approaches zero and the turbine comes to a stall. This is an interesting fault protection property, which is natural and inherent to an uncontrolled electric source. It is noticeable that the wind turbine generator’s V–I characteristics can be divided into two regions: a normal operation region (or a Z source region) and a fault protection region (or overload region). Therefore, an uncontrolled (or open-loop) wind turbine generator is a natural Z source with fault protection.

**Table 1:** Wind turbine and permanent magnet synchronous generator parameters^17,18^.

Wind turbine: manufacturer: AAER Canada; model: A2000-80; Rated Power: 2 MW; Rotor Radius: 40 m; swept area: 5027 m^2^		
Generator type	Non-salient pole PMSG, 2.0 MW, 690 V, 9.75 Hz,	
Rated mechanical power	2.0 MW	1.0 pu
Rated apparent power	2.2419 MVA	1.0 pu
Rated line-to-line voltage	690 V (rms)	
Rated phase voltage	398.4 V (rms)	1.0 pu
Rated stator current	1867.76 A (rms)	1.0 pu
Rated stator frequency	9.75 Hz	1.0 pu
Rated power factor	0.8921	
Rated rotor speed	22.5 rpm	1.0 pu
Number of pole pairs	26	
Rated mechanical torque	848.826 kN.m	1.0 pu
Rated rotor flux linkage	5.8264 Wb (rms)	0.896 pu
Stator winding resistance, *R*_*s*_,	0.821 mΩ	0.00387 pu
*d*-axis synchronous inductance, *L*_*d*_	1.5731 mH	0.4538 pu
*q*-axis synchronous inductance, *L*_*q*_	1.5731 mH	0.4538 pu

#### Natural (or passive) Z source example II: PV cells

**Figure 8:**
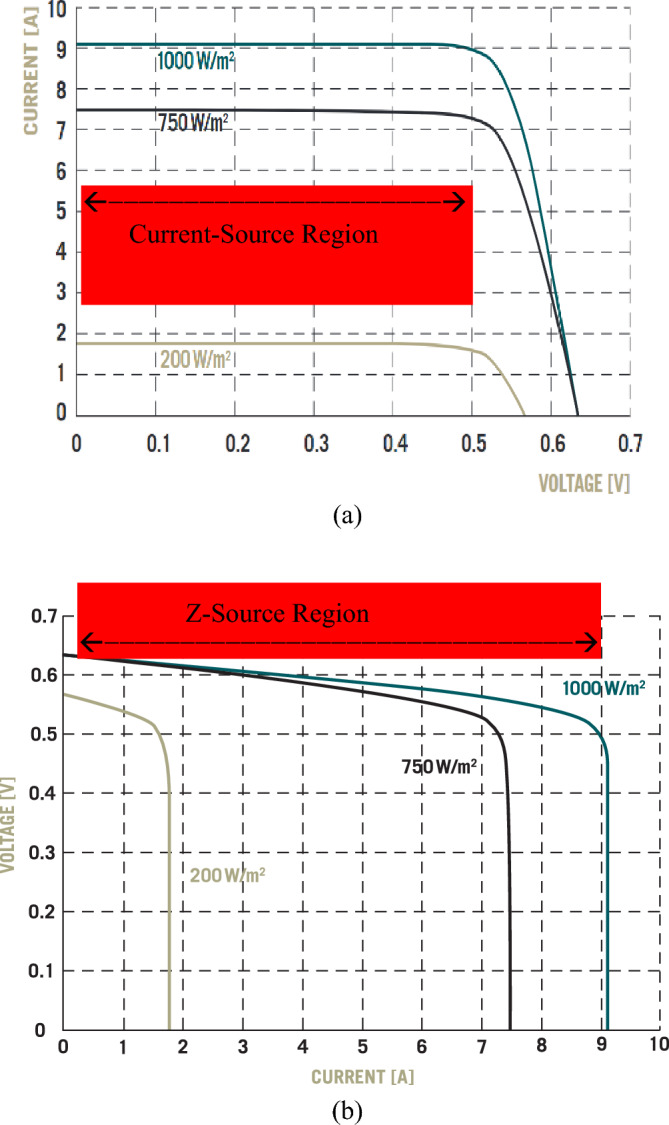
(**a**) I–V characteristic curves of a typical PV cell^19^. (**b**) Redrawn PV cell V–I characteristic curves from (**a**).

**Figure 9:**
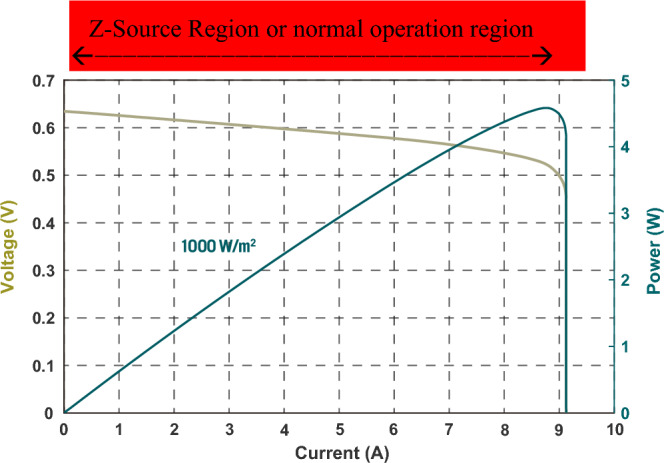
A specific V–I characteristic curve at 1000 W/m^2^ and its power–current (P–I) curve.

Figure 8a shows I–V characteristic curves of a typical photovoltaic (PV) cell. A PV cell when excited by constant intensity light is traditionally modelled as an ideal current source connected with an anti-parallel diode and a shunt admittance^7^. The curves do show a large area as a current source, however, redrawing the I–V curves to V–I curves as shown in Fig. 8b unveils a total different picture, that is the current-source region is very small and negligible. Moreover, a closer look into one particular V–I curve and its power–current (P–I) curve as shown in Fig. 9 reveals that a PV cell is a good example of a practical Z source as illustrated in Fig. 4 of the previous section, which consists of three regions: a small voltage-source region, a small current-source region, and a large Z-source region. More notably, a PV cell is normally not operated in its current-source region nor in its voltage-source region despite traditionally modeled as a current-source. Instead, it is normally operated in the Z-source region to produce the maximum or close-to-maximum power. In the Z-source region, the source impedance (or the source resistance in this DC case) exhibits a source resistance very close to its load resistance, that is, *Z*_*S*_ ≈ *Z*_*L*_, or *R*_*S*_ ≈ *R*_*L*_.

#### Natural (or passive) Z source example III: fuel cells

**Figure 10:**
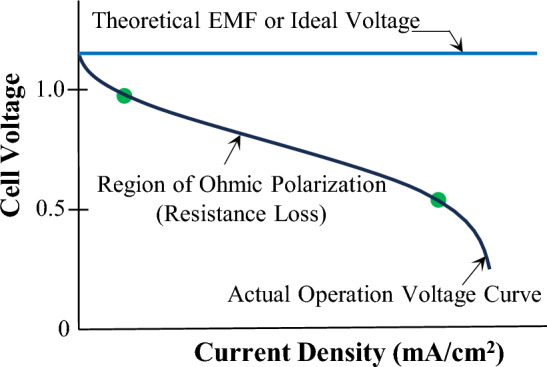
Typical fuel cell polarization curve.

Figure 10 shows a typical fuel cell polarization curve (or operation voltage curve or V–I characteristic curve)^8,9^. Clearly, a fuel cell far away from its theoretical or “ideal” voltage curve (the straight horizontal line) exhibits significant and strong dependency between output voltage and current like a Z source. Actually, its normal operation region, i.e., the Ohmic (or resistive) polarization region between the two dots as shown in the curve, is characteristically a Z source.

#### Artificial (or active) Z source example I: A dc–dc converter with output voltage/current control

**Figure 11:**
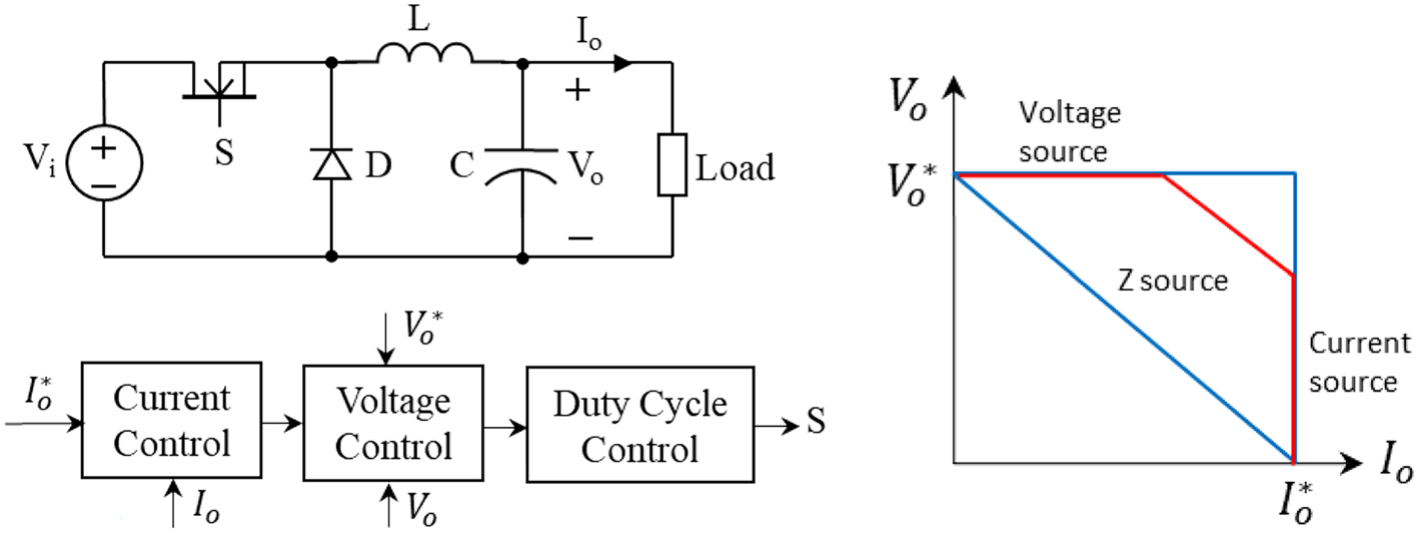
Dc–dc converter with output voltage/current (or resistance) control to behave like a voltage source (the horizontal line), current source (the vertical line), and a Z source/resistive source (a diagonal line).

Power electronics is a powerful enabling technology to implement artificial or active Z sources as well as to implement stiff voltage and current sources. A dc–dc converter can be controlled to have any desired output voltage/current relationship—literately, voltage/current equals to impedance or resistance—for example as illustrated in Fig. 11: controlling the converter to behave like a voltage source (*V*_*o*_ = *V*_o_^*^) for output current is less than its limit, *I*_*o*_ < *I*_o_^*^; like a current source when the current reaches to its limit, *I*_*o*_ = *I*_o_^*^; and like a Z source (or a resistive source) represented in diagonal lines if so desired. A dc–dc converter will have inevitable small power losses—that normally amount to less than 1% at utility-scale power ratings—in conduction voltage drop and switching power loss of the semiconductor switching devices. It is noteworthy, however, that no direct power loss is generated from the output voltage/current control. The Z source or resistive region as the voltage source and current source regions just has operational on-drop and switching losses. This output voltage/current control implemented by power electronics is, in fact, an active source impedance or resistance control with a resistance value comparable to its load resistance, which is, however, contradictory to traditional passive sources where a large internal passive resistance would generate large power losses. Therefore, a large internal resistance in a traditional power source is not practical when considering massive heat dissipation and low efficiency.

#### Artificial (or active) Z source example II: an inverter-based source with output voltage/current control

**Figure 12:**
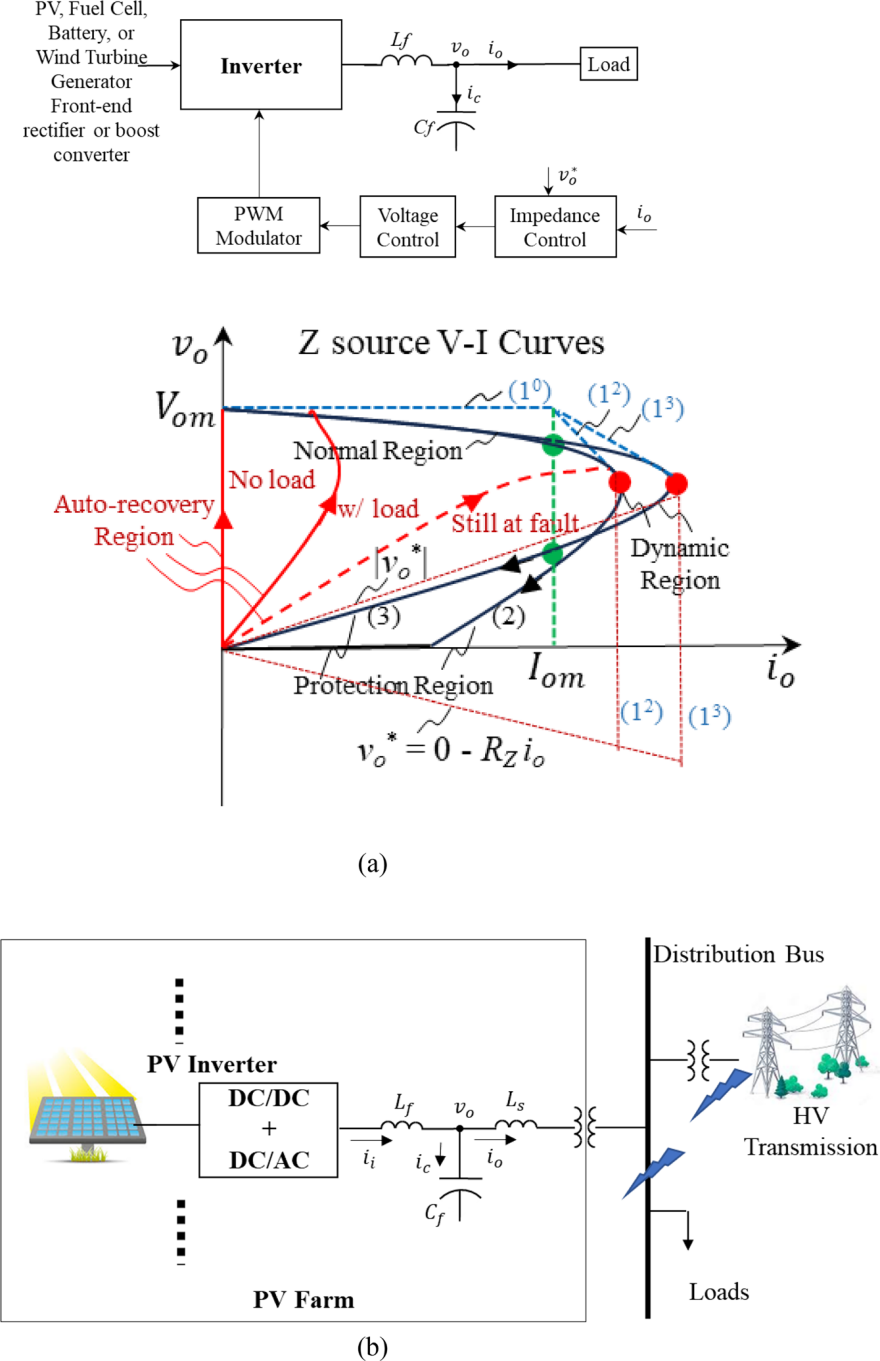

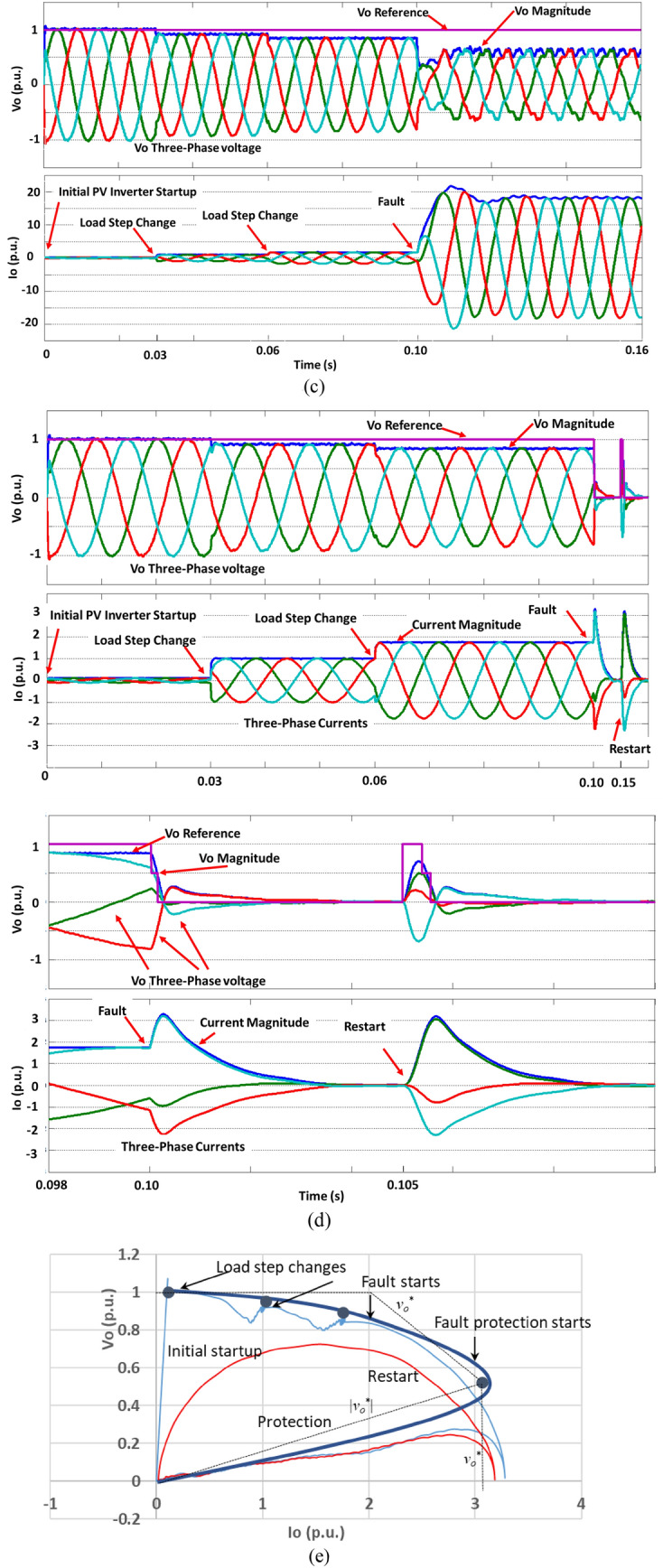
(**a**) A general inverter-based source is controlled to behave like an active Z sourcethat has four operation regions: normal operation, dynamic operation, protection, and auto-recovery operation, a so called “big nose” operation. (**b**) PV farm inverter configuration and assumed fault locations. Each PV inverter consists of an inverter bridge, LC filter (inductor *L*_*f*_ and capacitor *C*_*f*_), and output transformer to the local distribution and HV transmission line. (**c**) Simulation waveforms of the output voltage, *v*_*o*_, and current, *i*_*o*_, when the PV inverter is controlled as a stiff voltage source, including inverter startup, load step changes to 1 pu at time = 0.03 s and almost 2 pu at time = 0.06 s, and a fault at time = 0.1 s. (**d**) Simulation waveforms when the PV inverter is controlled as an active Z source. Top: entire waveforms including initial inverter startup, load step changes, and fault protection and restart attempt. Bottom: zoom-in waveforms of the fault dynamic operation, protection, and recovery attempt. (**e**) V–I trajectory curves of the PV inverter controlled as an active Z source. The blue curves are the trajectories before the restart (The fine trace: The bold blue curve: instant V–I curve including the initial inverter startup, load step changes and fault dynamic current limiting and protection, which contains switching ripples. The bold blue trace with dots shows the average values over one switching cycle, i.e., after filtering out the switching ripples). The red curve show the restart trajectory.

Similar to the above dc–dc converter, an inverter-based resource (or source) can be controlled like a Z source as shown in Fig. 12a. A Z source V–I curve has four regions: normal (with a stiff voltage reference of (1^0^), the flat blue dashed line), dynamic (with a reduced voltage reference like (1^2^) or (1^3^), the diagonal blue dashed lines), fault protection (with a voltage reference like a pure resistance (1^2^) or (1^3^), the red dashed lines), and auto-recovery operations (or regions). The dynamic region has multiple functions: ultrafast fault current limiting and handling transient current of dynamic loads. Different dynamic Z-source curves (or behaviors) can be utilized to meet different dynamic load requirements such as startups, inrush currents, fault clearances, etc. The stiff voltage and current sources (the horizontal and vertical lines of (1), respectively) are for steady-state operation boundaries, and a dynamic V–I trajectory as (2) or (3) could be governed according to tolerable temporary overcurrent magnitude and duration above the steady-state maximum current, *I*_*om*_. A criterion can be simply set according to an allowable peak magnitude of the current (ampere, *i*_*p*_), total charge (ampere-second, *i-T*), or total energy (ampere square-second, *i*^*2*^* − T*) during a transient period of *T*. When the dynamic operation cannot reduce the overcurrent to a satisfactory level, fault protection is kicked in with a voltage reference as (*v*_o_^*^ = 0 − *R*_*Z*_* i*_*o*_). Therefore, the inverter source becomes pure resistance to quickly reduce fault current and absorb energy that has built up in the circuit during the fault. The fault protection region is designed according to specific requirements such as the maximum allowable dynamic current and on how fast and how deep the final rest (or reset) point should be for the source. The fault protection region ends at the origin (zero voltage and current), after which an auto-recovery or re-startup of the source can be initiated. If the fault has been cleared, the source will start and follow one of the solid red lines depending on loading conditions. If the fault has not been cleared, the Z source will follow the dashed red line and will enter the over-current dynamic region again, which will trigger another round of protection. This auto-recovery function is one of the greatest feature of active Z sources, which will enhance resiliency in great deal.

#### Specific active Z source case study and simulation results

Following the above general description of the active Z source concept, a more specific case study and its simulations are provided in this subsection to verify the main features of active Z sources: such as ultrafast fault current limiting within microseconds (μs); protection and fault energy capturing, automatic recovery within milliseconds (ms); autonomous operation with no need for communication and artificial coordination. We have been examining three PV farms (20 MW, 40 MW, and 80 MW) all connected to a local utility grid for the US Department of Energy under two research contracts. Each PV farm has hundreds of PV inverters configured as Fig. 12b.

The main challenges for fault protection in today’s grids include slow response (typically 50 ms or longer) of circuit breakers, large time delay and latency in fault detection and communication, and imperfect artificial protection coordination. We can imagine that protection coordination among the above-mentioned massive number of PV inverters within each PV farm and among PV farms, and between PV farms and T&D networks is daunting, imperfect, or even impossible. Moreover, a short-circuit fault could trigger a catastrophic fire within a line cycle even before any traditional protection acts due to artificially regulated stiff bus voltages, unresponsive traditional power sources, and dominantly reactive circuits/networks that have inevitably stored up a large amount of energy, that all the three together powerfully feed a faulty circuit. The traditional power grid relies on the stiffness of bus voltages for grid stability and loadability because of its dominantly reactive sources, circuits, and networks.

We intend to use this specific case study and its simulations to show that the active Z source concept as the foundation have the great potential to solve the above fault protection problems. If each PV inverter is controlled simply like a traditional synchronous generator to provide a constant voltage, *v*_*o*_, a huge fault current is inevitable when a short-circuit fault happens at any point close to the PV inverter, near local loads, or at the high voltage transmission line as in Fig. 12b. Figure 12c shows simulation waveforms of a PV inverter output voltage, *v*_*o*_, and current, *i*_*o*_, including initial inverter startup, load step changes to 1 pu at time = 0.03 s and to almost 2 pu at time = 0.06 s (assuming that the maximum steady-state operation current is 2 pu), and a fault close to loads or at the high voltage lines happens at time = 0.1 s. The fault current rapidly rises up to 20 pu within one quarter line cycle (or < 5 ms), which eventually will trigger either PV inverter’s shut-down and/or circuit breakers. Traditional circuit breakers’ protection is too slow to prevent fires. And a simple shut-down of the PV inverter is not good either, although it is a traditional way to protect the PV inverter itself, but not the best way for system protection as a whole, because already highly charged transformers, inductors and capacitors in the circuit would keep feeding and releasing their stored energy to the faulty circuit—which may cause sparks and fires—even after the PV inverter has been switched off.

Now if each PV inverter is controlled like an active Z source illustrated in Fig. 12a with four operation regions, such huge fault current would never happen, as shown in Fig. 12d for the simulation waveforms, and Fig. 12e for their V–I curves and trajectories. The PV inverter is controlled as a stiff voltage source in the normal operation region when *i*_*o*_ < *I*_*om*_ (which is preset to 2 pu). When the current rapidly rises at a fault and exceeds its 2-pu limit, the PV inverter enters the dynamic region with reduced voltage reference to slow down and limit the fault current at an ultrafast speed within one inverter switching cycle (or microseconds. For our case, the switching frequency is 10 kHz or a 100 μs switching cycle. Within each switching cycle, there are 6 times of switching for a 2-level inverter and 12 times for a 3-level inverter). If the current still keeps rising and reaches its protection point (a preset value of 3 pu in the simulation), the PV inverter’s output voltage reference is commanded to zero minus virtual resistance drops, thus forcing it to behave like a pure resistor when viewed from the fault (or *v*_o_^*^ = 0 − (*R*_*i*_* i*_*i*_ + *R*_*Cf*_* i*_*C*_ ) = 0 − *R*_*Z*_* i*_*o*_) and the inverter enters the protection operation/region. As a result, the PV inverter successfully brings the fault current down to its 2-pu limit within 1 ms and further down to zero within 3 ms. It is noteworthy that commanding the output voltage reference to make the entire inverter system including the LC filter like a resistor and keeping the inverter running like a resistor are very critical and essential to reduce fault current quickly and at the same time to capture the already highly charged inductors, capacitors, and transformers in the circuit by back-feeding their energy to the inverter’s dc capacitors rather than letting those reactive (yet energy stored) components continue feeding the faulty circuit and cause sparks or fires.

Following the successful protection operation that has brought the output voltage and current down to zero, an recovery restart was initiated immediately (only 5 ms after the initial fault) as shown in the simulation. Since the fault has not been cleared, the attempt caused a rapid current rise and triggered another round of protection. However, if the fault had been cleared, the PV inverter would startup like the initial startup and restore the system. The simulation waveforms and actual operational V–I trajectories clearly demonstrated the main features of active Z sources: ultrafast fault current limiting within microseconds (μs); self-protection and fault energy capturing, automatic recovery within milliseconds (ms); inherent and natural self-protection with no need for communication and artificial coordination. As a result, the Z source control makes it possible to have resilient PV farms and fire-free faults.

## General discussions and methods/approaches

### Discussion and comparison of the voltage, current, and Z sources

In this section, we extend discussions to more broad and general principles, compare the voltage, current, and Z sources, and reveal some fundamental and yet interesting characteristics unique to each type of sources. In addition, we look back to the prior art and the origin of the Z source concept for deeper connection to its frequency-domain characteristics. The discussion and comparison will help us understand Z source’s uniqueness, potential and suited new system configurations, which will eventually lead us to new methods/approaches to engineering the future power grids from sources to circuits and systems.

#### Source-load connections: parallel or series?

**Figure 13:**
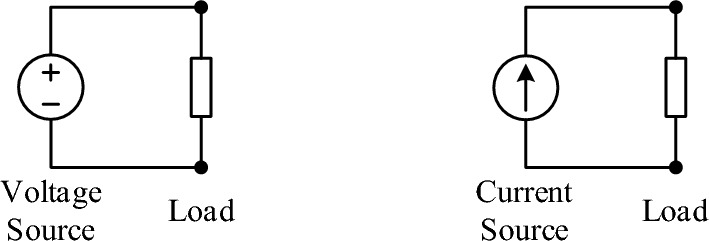
A single load-to-source connection.

When we connect a single load to a voltage source or a current source as in Fig. 13, “is the load in parallel or series with the source?” This question—as we often ask in our electrical and electronic circuits classes—may seem trivial or even nonsense. However, a close examination of these two simple source-load circuits and their “true” connections reveal an insightful nature, relationship, and property.

**Figure 14:**
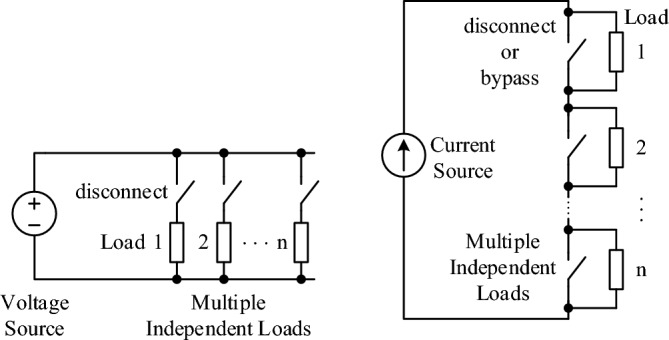
Multiple independent loads connected to a voltage source and a current source, respectively.

Our correct answers are that “a load is always —or at least should be connected—in parallel with the voltage source” and “a load is and should be connected in series with the current source”. Why? Consider connecting a second load or multiple loads to the source and each load with a disconnect switch—as we do in electricity grids—for independent switch-on and -off of individual loads. Figure 14 shows the source-load connections for the voltage and current sources, respectively. It is clear that we must connect loads in parallel with the voltage source and in series with the current source. Although we could connect two or more loads in series together and then as one load to a voltage source, however, the series-connected loads would have to share the voltage, cannot have its own disconnect switch, thus will not be independent to each other. Similarly, we have the dual conclusion for the current source. More interestingly, the disconnect switch must be in series with each load for the voltage source-load connection, whereas it must be in parallel with each load for the current source-load connection—maybe it should be called “bypass switch” for current-source loads.

Theoretically and advantageously, both the parallel and the series source-load connections are operable for a Z source. This may yield many interesting applications that are suited for Z sources.

#### Suited sources, loads, and applications

A voltage source is suited for relatively high impedance loads with low current and high voltage such as household electric appliances, whereas a current source for relatively low impedance loads with low voltage and high current such as electronic loads, data center loads, CPU chips, where each load is rated at low voltage (1.5—12 Volts) and high current (10 s—100 s Amps). A good application may be wired and wireless charging of electronic devices in series by a single current source.

Most loads today are made as voltage-fed regardless of their suitability. However, the above-mentioned relatively low voltage and high current loads can be—and should be made— as current-fed to fully utilize advantages of current sources.

It should be noted that the Z source offers the above features of both voltage and current sources, thus is suited for almost all the above applications. Multiple Z-source type of power sources and/or loads can be connected in series, parallel, and/or a combination of series and parallel to achieve maximum system-level flexibility and efficiency. In addition, how to use the Z source in electricity grids as a whole will be explored in the next section.

#### Properties and dual relationships

The dual circuit of a voltage source is a current source and vice versa. However, an ideal voltage source can never be replaced by an ideal current source or vice versa. The dual circuit of an impedance source is an admittance source. Admittance is the reciprocal (or inverse) of an impedance—reciprocal mathematical expressions of a circuit’s voltage-current relationship—therefore, the dual circuit of an impedance source is physically itself with its reciprocal expression—and so is an admittance source. It is interesting to note that an ideal Z source—when its source impedance (or admittance) matches its load impedance (or admittance), that is *Z*_*S*_ = *Z*_*L*_,—delivers the maximum signal, power, or energy from the source to load. An ideal voltage source or ideal current source does not have this property.

#### Prior Z-source work and applications

Although the Z-source models presented in this article have not been found in the literature, the Z-source concept has been used in power converters and inverters^10,11^. Traditionally, a power converter or inverter is fed by either a voltage-source or a current-source. A voltage-source inverter is not allowed to operate at any shoot-through (or short-circuited) switching states that could destroy and explode the inverter. In its dual circuit fashion, a current-source inverter is therefore not allowed to operate at any open-circuited switching states that could destroy and explode the inverter as well. These forbidden short-circuited or open-circuited switching states have been the detrimental reliability problem for power conversion technology due to high speed switching and mis-triggering from EMI noises. Passive Z-source circuits/networks that consist of inductors (L) and capacitors (C) or distributed LC networks like power cables have been originally applied to power conversion^10,11^ to solve the detrimental reliability problem, and to actively utilize the traditionally forbidden short-circuited (and open-circuited) switching states for voltage/current step-up or step-down operations with one single stage of power conversion.

The above-mentioned Z-source inverters/converters are implemented by inserting a passive LC network between the source and semiconductor switches to transform the original voltage (essentially capacitive) or current (essentially inductive) source into a Z source that exhibits a comparable impedance at very high frequency (10 s—1000 s of kHz) for a very short period of time (microseconds) to make use of those forbidden short-circuited and/or open-circuited switching states without destroying or exploding the inverter. Therefore, passive LC (or Z-source) networks have limited applications to power conversion circuits. The Z-source models presented in this article, however, have expanded the Z-source concept to a new level—the fundamental V–I characteristics in both time and frequency domains—that includes any frequency (from DC to high frequency) and any length of time period (from microseconds to minutes) for electricity grids—a totally new application field beyond power conversion—that will be presented in the next section as our methods/approaches to resilient and fire-free electricity grids.

### Implementing naturally balanced, stable, and resilient electricity grids

#### The root cause of power instability and congestion of today’s grids and a natural solution

**Figure 15:**
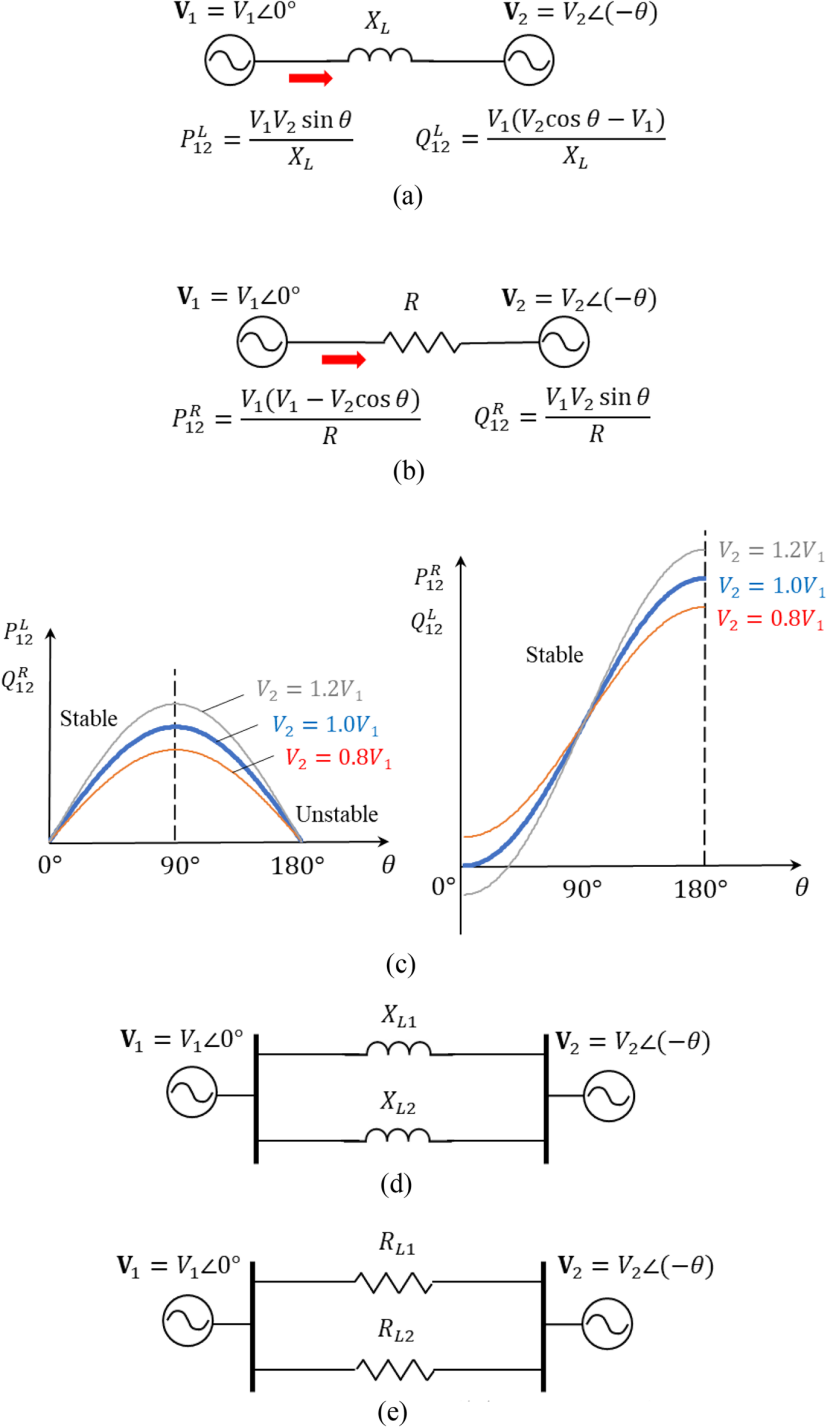
The root cause and a natural solution of power instability and congestion. (**a**) An inductive (or inductance-dominant) two-machine system—a power grid in its simplest form—and its power flow from bus 1 to bus 2. (**b**) A Resistive (or resistance-dominant) power grid. (**c**) Power Curves of inductive and resistive power grids. (**d**) Two inductive parallel transmission lines. (**e**) Two resistive parallel transmission lines.

Figure 15a illustrates the classical power grid instability problem^12,13^: a two machine (**V**_1_ and **V**_2_) system connected by an inductive (or inductance-dominant) impedance, *X*_*L*_ delivers power from bus 1 to bus 2 with the governing active and reactive power equations of $P_{12}^{L}$ and $Q_{12}^{L}$, and plotted curves in Fig. 15c. The active power curves as plotted in Fig. 15c exhibit two regions—stable and unstable—separated at the phase angle difference of 90°. Consider, however, a resistive power grid as shown in Fig. 15b, the active power curves become monotonically increasing over the entire range of 0 to 180°. A resistive circuit or system is simply physically stable by its nature. This natural solution is advantageous in terms of controllability and resilience, however, such resistance-dominant systems would be inefficient and lossy and thus have been avoided by all means in traditional power grids.

The root cause of power congestion and a natural solution are further illustrated in Fig. 15d,e. The power flow sharing between two inductive transmission lines is determined by their relative reactances, which differ from their thermal limits by their resistances. However, two resistive (or resistance dominant) transmission lines—if ever possibly engineered as illustrated in Fig. 15e—do not have such congestion or imbalance problem. They naturally balance out according to their resistance or thermal limits when their inductances are negligible and resistances dominant. It is true and natural that for DC power transmission, two parallel power lines would never have power sharing, balance, or congestion problem because they are purely resistive and their powers balance out naturally by their thermal resistances.

However, it was impossible to make long-distance AC transmission lines dominantly resistive, unless large resistors were physically inserted into the lines—which is not practical because of power losses. Fortunately, with today’s power electronics and our proposed approach, it becomes possible to implement dominantly resistive power lines (or resistive power grids as a whole) without such power losses from physically inserted resistors, thus will not sacrifice efficiency for grids. In the next subsection we describe how to make grids resistive or resistance-dominant as our final goal to make grids naturally balanced, stable, and resilient by using power converters/inverters as virtual/active resistors to transform grids to dominantly resistive systems.

#### How to make grids resistive or resistance-dominant

**Figure 16:**
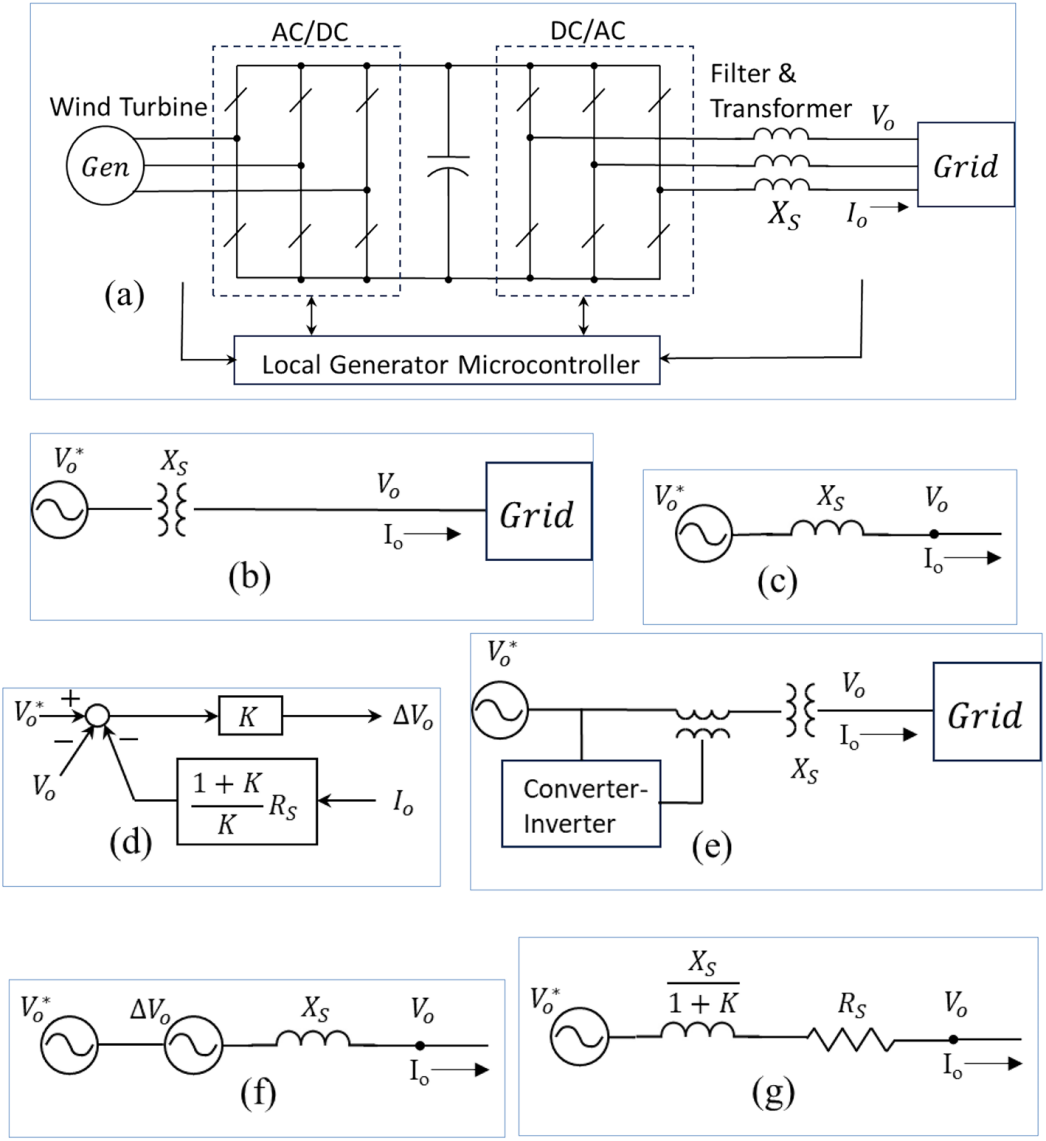
Adding power electronics and controls to transform an inductive (or reactance-dominant) grid to a resistive (or resistance-dominant) one^20^.

Power grids are mostly inductive. Figure 16 illustrates how to transform an inductive source or circuit into a resistive one. As examples, Fig. 16a shows a full power converter wind turbine generator and (b) a grid bus from a transmission or distribution substation. These originally inductive circuits or sources from an inverter-based resource or a transformer like Fig. 16a,b can be equivalently represented as Fig. 16c—an ideal voltage source with an inductive source impedance *X*_*S*_. Adding a simple control like Fig. 16d to the wind turbine generator’s microcontroller or adding a fractionally rated small power converter/inverter in series with the grid circuit/component as Fig. 16e^14^ with the control of Fig. 16d will result in an equivalent controllable source, Δ*V*_*o*_ as illustrated in Fig. 16f. Consequentially, the original inductive source and circuit become dominantly resistive as illustrated in the resultant equivalent circuit of Fig. 16g. Because the power electronics and control literally minimize the original inductance to a negligible level by a factor of (1 + *K*) and artificially insert a resistor (a virtual resistor), *R*_*S*_, to the circuit. This virtual resistor implemented by power electronics and control introduces no power loss from the resistance while providing the desired damping and stabilization functions for the circuit, system, and grid.

#### Nature-mimicking method/approach to achieve naturally balanced, stable, and resilient grids

We summarize our nature-mimicking method or approach as two steps: (1) making all sources fault protected Z sources and (2) making all grid circuits resistive or resistance-dominant—both by today’s enabling technologies: power (or energy) electronics, micro-electronics, information technology, and system control engineering. These technologies do not compromise efficiency while achieving fault protection and resiliency to extreme conditions like short-circuit or open-circuit caused by natural disasters or human-made events. As a result, fault protected and resilient electricity grids become possible and this most complex machine ever engineered and made over the last century by human beings—the electricity grid—would be transformed into a new future smart and resilient machine.

#### Putting all the pieces together to the big picture: resilient and fire-free grids

Thus far, we have described the Z source concept of electricity and its main features, how to make each power source an active Z source as shown in Fig. 12, and how to make grid circuits/networks dominantly or purely resistive as shown in Fig. 16. At the grid network level, the Z source concept and its active resistive control can be further extended to more advanced series compensation and control devices such as transformer-less unified power controllers, reactance cancellers, and active resistors, and hybrid transformers (that is, a combination of traditional power transformer and inverter)^14^.

Our big picture is resilient and fire-free grids: resiliency and fire-free fault protection. Resiliency means fast restoration/recovery after a fault. As shown in Fig. 12, an immediate recovery attempt can be initiated after each fault protection operation. If the fault has been cleared or isolated naturally like a flying debris or by traditional circuit breakers—it is true that we still need the traditional circuit protection for the grid—then PV inverters should be re-started. In order to have a fast recovery, we have to answer many remaining questions: instant startup of each PV inverter, instant synchronization with the grid, transient-less energization of transformers, loads, and networks, and instant black-start of bulk power systems. The Z source concept and resistive control technology have the potential to make them happen and some preliminary results has been shown in Reference^15^.

**Figure 17:**
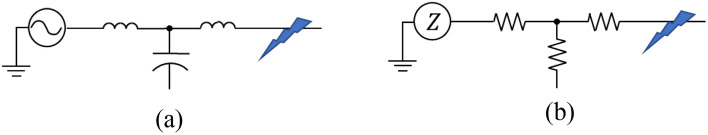
Equivalent circuits of Fig. 12b: (**a**) when the PV farm and the HV transmission line are controlled as a stiff voltage source. Even when the source is shut-down or reduced to zero volt, the fully charged filter and transformer inductors and capacitors would keep feeding the fault and cause fires; (**b**) when the PV farm and the HV transmission line are controlled as a Z source and resistive circuit. The PV inverter can virtually place a virtual resistor in each of the three branches (or the *L*_*f*_, *C*_*f*_, and *L*_*S*_ branches), respectively, thus making the source and circuit purely resistive. With a zero-volt source and purely resistive circuit, the fault will not get any energy any longer.

The other half of our big picture is fire-free fault protection. Can a fire-free grid really possible? Consider that a 20-MW PV farm is connected to a 500-kV transmission line. If a tree branch connects with the power line at that high voltage and with that much power on it, it would likely cause a fire in any traditional power grid. Figure 17a shows an equivalent circuit of our investigated 20-MW PV farm Fig. 12b. Mainly because (1) the 500-kV line is controlled/regulated to a stiff voltage source by all the PV inverters and/or transmission substation control devices and (2) all the reactive components and circuits in the grid from each PV inverter all the way to the 500-kV line (i.e., filter inductors and capacitors, HV transformers with their leakage inductance and parasitic capacitance, transmission line inductance and capacitance, and so on) are highly charged and they store a great amount of energy, especially exacerbated during a fault would feed the faulty line even when the PV farm has been switched off from the line. For example, a high voltage transformer after being switched off can hold charges or high potentials for hours by its insulation and could cause sparks and fires when accidentally shorting their terminals. As we have discussed previously, a highly charged capacitor is a voltage source that cannot be shorted without causing stress to the circuit and a highly charge inductor is a current source that cannot be opened. Any faults to short a highly charged capacitor or to open a highly charged inductor would likely cause sparks and fires.

However, the presented Z source concept and resistive control technology can change the story. If we control all PV inverters as a Z source (i.e., a pure resistor during a fault as in subsection *6) Specific Active Z Source Case Study and Simulation Results*) and make/transform all components and circuits to resistive elements by strategically positioned power electronics (converters and inverters) control devices in the grid, the PV farm and transmission line shown in Fig. 12b would become Fig. 17b, pure resistive circuit during the fault protection, that is, all the ac components and circuits become resistive and grounded through the inverters, thus has no energy to the fault. During this fault protection, each PV inverter is running at its full capacity with full or even more dc voltage to keep absorbing energy stored in the circuits and making them like a resistor. All the energy originally stored in the reactive components will eventually be diverted and discharged to the dc capacitors within PV inverters. As a result, the Z source not only limits fault current within a preset value that is 10 times less than a traditional circuit breaker’s interrupt current but also acts at a ultrafast speed (< 3 ms, that is more than 10 times faster traditional circuit breakers that have to wait for zero crossings of their line currents) to bring the fault current down to zero, which hopefully will make the grid fire-free.

## Summary, conclusion, and future work

Electricity infrastructure (i.e., the power grid) is the greatest engineering achievement of the twentieth century, according to a U.S. National Academy of Engineering report^16^. The traditional power grid has been engineered and constructed over more than a century, is aging. Old power plants and their technologies are obsolete. Our modern societies would not survive without electricity on one hand, however, on the other hand electrical faults could cause and have caused many catastrophes—mainly deadly fires—to our societies.

In summary, we have identified that the root cause for electrical fires is because power grid sources have been all traditionally engineered and controlled to behave like an ideal voltage source, which is ideal for loads on one hand, however, unintentionally and catastrophically feeding faulty circuits on the other hand.

In our research findings of this article, we have shown that most renewable energy sources without feedback control of their output voltage and/or output current are actually a natural (or passive) Z source of electricity, which has self-protection and fault-current limiting functions. Moreover, with power electronics and active control, we can make any power source to an active Z source, and the entire grid circuits to ideally resistive, which in turn results in ultrafast, inherent and natural self-protection, fault-current limiting, auto-recovery, and therefore potentially fire-free grids resilient to natural disasters and human-made events.

In this article, we have shown the methods and approaches and their great potential to revolutionize today’s power grid. However, many questions and research topics remain, which require many engineering communities—power electronics, power system engineering, controls, and so on—working together to solve. Future work includes the following topics as examples:

A. Bulk power grid simulation and modeling that implement the methods described in the article from Z-source power generation to distribution and transmission network transformation from doninanlt reactive to resistive and their comparisons to the traditional grid implementations.
B. Modeling of hybrid AC and DC grids with mixed parallel and series system configurations/connections on both sources and loads as discussed in Section IV Discussion and Comparison of the Voltage, Current, and Z Sources to explore Z-source’s unique features that neither voltage-nor current-source has.
C. Development and verification of active Z-source control models (or different V–I characteristic curves for different load and system requirements) to achieve natural and instant fault-current/fault-voltage limiting without the need for faster protection in settings of large number of Z sources and dominantly resistive circuits.

### Supplementary Information

Supplementary Information.

## Data Availability

The datasets used and/or analysed during the current study available from the corresponding author on reasonable request.
